# Virgin Olive Oil By-Product Valorization: An Insight into the Phenolic Composition of Olive Seed Extracts from Three Cultivars as Sources of Bioactive Molecules

**DOI:** 10.3390/molecules28062776

**Published:** 2023-03-19

**Authors:** Lorenzo Cecchi, Giulia Ghizzani, Maria Bellumori, Carmen Lammi, Bruno Zanoni, Nadia Mulinacci

**Affiliations:** 1 Department of Agricultural, Food and Forestry Systems Management (DAGRI), University of Florence, Piazzale Delle Cascine 16, 50144 Florence, Italy; 2 Department of Neuroscience, Psychology, Drug and Child Health, Pharmaceutical and Nutraceutical Section, University of Florence, 50019 Florence, Italy; 3 Department of Pharmaceutical Sciences, University of Milan, 20133 Milan, Italy

**Keywords:** nüzhenide, nüzhenide 11-methyl oleoside, oleuropein, olive ripening, Tuscan varieties, *Olea europaea* L., Frantoio, Moraiolo, Leccino, nutraceutical molecules

## Abstract

Olives are very rich in phenolic compounds with important health-promoting properties. The profile and content of phenols in olive pulp and virgin olive oil are strongly influenced by the fruit ripening degree, but little is known concerning the evolution of phenolic compounds in the seed. In this work, the phenolic composition of seed from Tuscan cultivars (Frantoio, Moraiolo, Leccino) was studied over maturation. Starting from each seed sample, a phenolic extract was prepared and analyzed by HPLC-DAD-MS. Nüzhenide and nüzhenide 11-methyl oleoside were by far the most abundant phenolic compounds; their content reached up to 46 g/kg in dry seeds, although this diminished in the final stage of fruit maturation. At the same time, the phenolic composition of the pulp was also characterized over the course of maturation, showing that oleuropein was by far the most abundant compound, with concentrations comparable to those of nüzhenide and nüzhenide 11-methyl oleoside in the seeds. Overall, the total amount of phenols in seed dry extracts was significant, reaching approx. 100 g/kg. The chemically characterized dry phenolic extracts from seeds could be used for future biological assays aimed at evaluating the potential bioactivities of these phytocomplexes.

## 1. Introduction

In recent years, much evidence has supported the important health beneficial effects of the phenolic compounds present in extra virgin olive oil (EVOO) and in related by-products [1,2,3]. These compounds are mainly constituted by the secoiridoid derivatives, a class of phenolic compounds which is typical of *Olea europaea* L., but also by lignans and lower amounts of flavonoids, phenolic acids and phenolic alcohols [4,5,6,7,8]. Due to the well-documented effects against inflammation, diabetes, cardiovascular and neurodegenerative diseases exerted by the different components extracted from different parts of the olive tree [9,10], the production and characterization of such extracts is required [11]. Among the different types of secondary metabolites from the olive tree (e.g., sterols, phenolic compounds, tocopherols, pigments, triterpenoids, hydrocarbons), phenolic compounds are the most widely studied, as they have shown the most promising health-promoting effects. This was clearly confirmed by observations of the in vivo protection from oxidative damage of low-density lipoproteins (LDL) exerted by olive oil phenolic compounds [12,13], leading the European Food Safety Authority (EFSA) to approve a health claim for olive oil polyphenols [14]. Other well-recognized health benefits attributed to phenols from *Olea europaea* L. have been described in the literature as antioxidant and anti-inflammatory properties, as well as the abilities to help in maintaining low levels of cholesterol, normal blood pressure and normal gastrointestinal tract function and to strengthen the immune system [15]. 

In the context of the circular economy, based on the concept of re-using and valorizing by-products which are therefore no longer considered as waste materials, recovering value from the various olive oil production by-products is receiving more and more attention [9,16,17]. According to the “zero waste” model, each residue of the olive oil production chain should be treated for use in traditional or innovative applications, e.g., as an energy source, compost, cosmetic, animal feed or nutraceutical ingredient for human consumption [9,17].

The olive fruit is constituted by an exocarp (i.e., the skin), a mesocarp (i.e., the pulp) and an endocarp (i.e., the pit) which is, in turn, constituted by the woody shell which encloses the seed [18]. The woody shell and the seed can be considered as a by-product of the production of VOO from de-stoned olives, but also of the pitted table olives [19,20]. It has been reported that specific machines are now available which are able to recover the entire seed from the whole endocarp after de-stoning olives [21]. The woody part is quite poor in phenolic compounds, and it is mainly used as a source of energy, while it has been reported that the seed is rich in phenolic compounds [22]. 

Concerning seed components, some pioneer studies have highlighted beneficial healthy effects exerted by proteins extracted from olive seeds [16]. Other studies in the literature have shown some preliminary health-promoting effects exerted by nüzhenide, one of the main phenolic components in the seed [23], or by phenolic extracts from olive seeds [19]. However, in order to highlight the possible advantages in recovering phenols from olive seeds, further detailed studies on their composition and biological activities are required. 

In this sense, the first step is the acquisition of deeper knowledge of the phenolic profiles of olive seeds and the influence of the variety and ripening time. To date, only a few manuscripts in the literature have described the phenolic composition of *Olea europaea* L. seeds. Overall, it has been reported that the main phenolic compounds present in the seeds are bitter glucosides such as nüzhenide and nüzhenide-11-methyl oleoside. These molecules bear in their chemical structure at least two glucose moieties, which make them poorly liposoluble, and consequently, they are not extracted into the olive oil [21]. However, some authors have reported the presence of nüzhenide but not of nüzhenide-11-methyl oleoside [11] while detected observed neither nüzhenide nor nüzhenide-11-methyl oleoside in olive seeds [24]. Furthermore, some studies have reported a clear prevalence of phenolic compounds in the glycosylated form [19,21,25], while other authors have reported the presence of phenolic compounds other than secoiridoids and in non-glycosylated forms [24]. From a quantitative point of view, to the authors’ knowledge, there is a lack of data in the literature. In one manuscript, the authors only reported a total phenolic compounds content of 2.79 mg/g seeds, with no specification of the method used for quantitation and with no details on the amount of each phenol [21]. In another work [19], only six molecules were detected, and these seemed to be the molecules that are typically found in the olive pulp rather than those associated with olive seed (the only exception was nüzhenide). In the work of Elbir et al. [25], the total phenolic content of the whole stone was reported, albeit showing only the percentage chromatographic areas of the single detected phenols. In a study of the seeds from the olives of six Portuguese cultivars, considered at the optimum ripening degree according to the skin color, the secoiridoids extracted from seeds were identified as an oleuropein equivalent, i.e., nüzhenide 11-methyl oleoside was the most abundant compound in the Lentisca cultivar (reaching 16.1 g_ole_/kg), followed by nüzhenide (12.2 g_ole_/kg). The same work reported that olive seed phenols were almost all secoiridoids, with the exception of tyrosol [26]. These literature data make it necessary to clarify the composition of this part of the olive fruit. The phenolic composition of the olive seed is certainly of interest in efforts to define possible uses of this by-product. In the literature, only the phenolic compositions of olive seeds from a handful of different samples have been described, and no study has reported the evolution of the phenolic composition of olive seeds over the course of maturation of the fruits of different cultivars. To the best of the authors’ knowledge, only one study has examined the qualitative phenolic composition of olive seeds at different ripening stages, but no quantitative data were provided [22].

With this in mind, the aim of this research was to determine the phenolic composition of the olive seeds of three Tuscan cultivars (i.e., Frantoio, Moraiolo, Leccino) over the course of maturation. To this end, fruit samples of the three cultivars were collected at different levels of ripeness in the period of 15 September–17 November 2020. Dry phenolic extracts of the seeds were produced, allowing us to determine the final yields and the phenolic profiles by HPLC-DAD-MS. Bearing in mind the possible uses of the dried extracts from seeds for future biological assays, the best mode of quantitation is also discussed. 

## 2. Results and Discussion

The study mainly aimed to provide a detailed characterization of the phenolic profile of olive seeds, also evaluating its evolution during maturation in three typical Tuscan cultivars: Frantoio, Moraiolo and Leccino. The importance of this type of characterization is underlined by the well-documented health-promoting properties of the phenolic compounds in *Olea europaea* L.; these properties have already been widely investigated for the phenols from olive fruit pulp, virgin olive oil, olive leaves and olive oil production by-products [9], but to date, they have not been thoroughly investigated for the phenols from the olive seed. As a further objective, we wanted to compare the phenolic concentrations in seeds with those in the whole fruit in order to provide useful information about the advantages of preparing phenolic extracts from the seed with respect to the whole fruit and to provide useful information for the use of the pit as a source of specific phenolic components. The steps toward these objectives have been:

- collecting samples of olive fruits of the three cultivars to evaluate the biodiversity and evolution during ripening of the phenolic contents of seeds;
- characterizing these samples in terms of yield with respect to the weight of the different parts of the fruit (Table 1);
- defining a protocol for the extraction of phenols from olive seeds that enables both the analysis of phenols by HPLC-DAD-MS and the preparation of a dried extract suitable for future biological assays;
- characterizing the phenolic extracts of the seed from both qualitative and quantitative standpoints;
- characterizing the phenolic profile of the pulp and comparing the results with those previously reported in the literature, and with those for seeds.

### 2.1. Characteristics of the Collected Olive Samples

The olive fruit samples of the three Tuscan cultivars were collected at seven sampling dates from September to November. Table 1 reports the composition of the fruits in terms of whole fruit weight, seed weight (also as % with respect to the whole fruit), pulp/stone ratio and moisture of the whole fruit. The table also reports the yield % of the phenolic extracts obtained from the seed samples.

Overall, the weight of 100 whole fruits increased over the course of ripening for all three cultivars, passing from 101.5 to 166.6 g (+64%) for the Frantoio cultivar, from 108.6 to 159.2 g (+46%) for the Moraiolo cultivar and from 106.0 to 136.2 g (+28%) for the Leccino cultivar. Concerning the data of the Leccino cultivar, the datum related to the sixth sampling point appears to be an outlier. The weight of the 100 seeds (which were those from the same 100 whole olives) showed different behavior for those of the three cultivars: it increased from 2.195 to 4.323 g (+97%) for the Frantoio cultivar, and from 2.515 to 4.418 g (+76%) for the Leccino cultivar. On the other hand, for the Moraiolo cultivar, it did not increase, passing from 3.150 g at the first sampling to 3.082 g at the last sampling, and fluctuating from a minimum of 2.790 g to a maximum of 3.395 g. Of course, this different behavior of the seed weigh resulted in a different behavior of the seed % weight, which showed an increasing trend for the Frantoio and Leccino cultivars and a decreasing trend for the Moraiolo cultivar. As for the pulp/stone ratio, an increasing trend was observed for the three cultivars, with comparable values for Frantoio (from 1.62 to 2.98) and Leccino (from 1.75 to 2.84) and higher values for Moraiolo (from 2.38 to 3.95). Finally, the moisture content increased for the three cultivars, albeit with slightly different trends: for Frantoio, it increased from 44.7% to 57.6%, with the highest increases observed at the second, sixth and seventh sampling points; for Moraiolo, it increased from 48.1% to 54.8%, with the increase having already largely occurred by the second sampling point (i.e., 53.3%); similarly, for Leccino, it increased from 48.0% to 56.8%, with the increase having similarly almost completely occurred by the second sampling point (i.e., 56.0%). 

### 2.2. Preparation of the Dry Phenolic Extracts from the Olive Seeds

**Figure 1:**
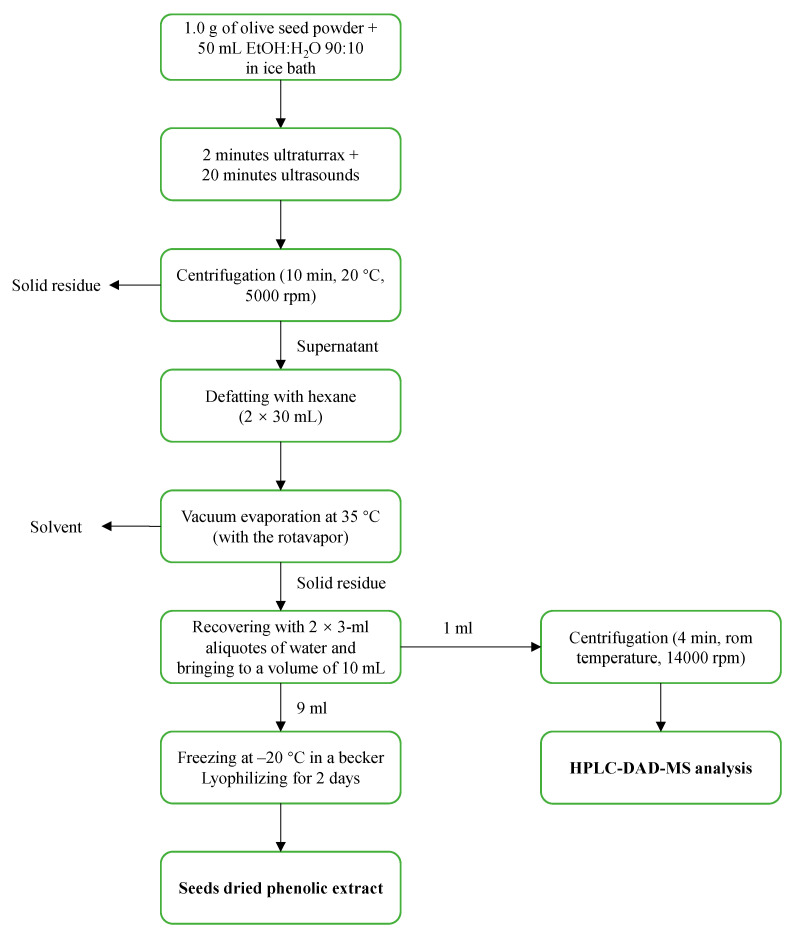
Procedure for the preparation of dry phenolic extracts from seeds.

**Figure 2:**
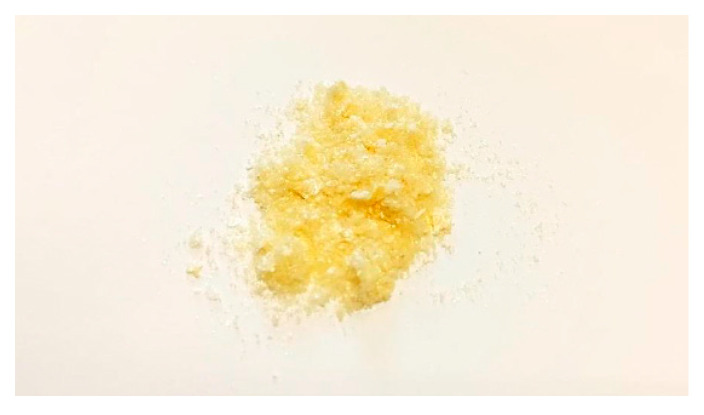
Dried phenolic extract from the seeds of the Moraiolo cultivar at the fourth sampling point.

In order to prepare a phenolic extract from the seeds which would be useful both for HPLC analysis and for future biological tests, we defined a protocol, as illustrated in Figure 1. After some preliminary extraction trials, performed in a previous study, the EtOH:H_2_O 90:10 extractive solution was selected (this gave the same extraction yields as using lower percentages of EtOH (e.g., 70%), but the samples were more easily and quickly concentrated during vacuum evaporation), with an extractive ratio of 50 g of extractive solution for 1 g of the seed powder prepared as described in Section 3.3. In order to reduce the extraction time and to maximize yield, after some preliminary trials, the combined use of the Ultraturrax (2 min) and of the ultrasounds bath (20 min) was selected as the best extraction approach. After the separation of the liquid extract from the solid residue by centrifugation, the supernatant was defatted twice with *n*-hexane to remove any fatty residue, as this would hinder the solubility of the sample in the aqueous media commonly used for biological assays. In the successive step, the defatted extract was vacuum dried and recovered with water to a total of 10 mL. The aqueous phenolic extract so obtained was split into two aliquots: a first 1-mL aliquot to be used for the HPLC-DAD-MS analysis after centrifugation at 14,000 rpm, and a second 9-mL aliquot which was lyophilized, thus yielding a light-yellow dried extract (Figure 2). The dried extracts were easily pulverizable and maintained the same characteristics for at least 12 months after lyophilization, confirming their storability. 

### 2.3. Characterization of the Phenolic Profile of Olive Seeds over the Course of Ripening

**Figure 3:**
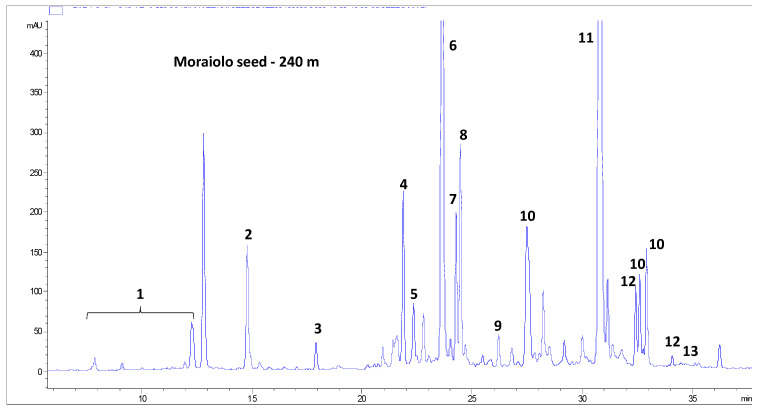
Chromatographic profile at 240 nm of the phenolic compounds in seeds of the Moraiolo cultivar at the fourth sampling date. 1, tyrosol derivatives; 2, oleoside 11-methyl ester; 3, oleoside 11-methyl ester isomer; 4, nüzhenide derivative; 5, verbascoside; 6, nüzhenide; 7, bis(oleoside 11-methyl ester) glucoside; 8, salidroside oleoside; 9, nüzhenide isomer; 10, nüzhenide di-(11-methyl oleoside) isomers; 11, nüzhenide 11-methyl oleoside; 12, nüzhenide di-(11-methyl oleoside) isomer 1; 13, nüzhenide di-(11-methyl oleoside) isomer 1; 14, ligstroside oleoside.

Table 2 reports the evolution over the course of ripening of the content of the molecules identified in olive seeds of the three cultivars, Frantoio (Table 2A), Moraiolo (Table 2B) and Leccino (Table 2C), based on UV and mass spectra analyses and literature data. An example of a chromatogram of the phenolic profile of seeds of the Moraiolo cultivar is given in Figure 3. Overall, 19 molecules were tentatively identified, 16 of which bore at least one phenolic moiety in their chemical structure. The remaining three molecules (i.e., oleoside 11-methyl ester, oleoside 11-methyl ester isomer and bis(oleoside 11-methyl ester) glucoside) are constituted by residues of glucose and elenolic acid, typical non-phenolic residues of the secoiridoids phenols from *Olea europaea* L. Interestingly, the phenolic moiety of 15 out of the 16 phenolic molecules identified (i.e., all the tyrosol derivatives, including salidroside oleoside, nüzhenide and its derivatives, and ligstroside oleoside) was tyrosol, while only in the case of verbascoside was it hydroxytyrosol. This situation is very different for the other products from *Olea europaea* L. (e.g., olive fruit pulp, virgin olive oil, olive leaves and virgin olive oil production by-products), in which hydroxytyrosol derivatives usually prevail, or are at least present in comparable amounts with tyrosol derivatives [9].

**Figure 4:**
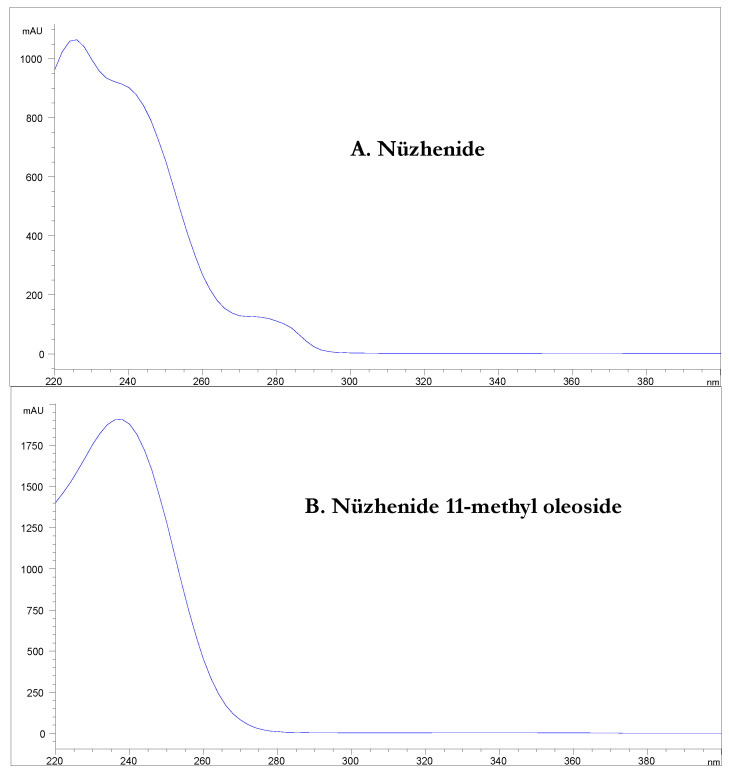
UV-Vis spectrum of (**A**) nüzhenide and (**B**) nüzhenide 11-methyl oleoside.

For all the three cultivars, two phenolic molecules were largely prevalent in the phenolic profile of the olive seeds: nüzhenide and nüzhenide 11-methyl oleoside. This result is in agreement with the literature [22,23,24,25]. The UV spectrum of these molecules presents a high absorption at 240 nm but a low absorption (or no absorption, as in the case of nüzhenide 11-methyl oleoside) at 280 nm (Figure 4). 

For this reason, in order to be as reliable as possible, the quantitation of nüzhenide and its derivatives was performed using the calibration line built with the nüzhenide commercial standard at 240 nm. To the best of the authors’ knowledge, this manuscript is the first report in which the typical nüzhenide derivatives present in the olive seeds of specific cultivars over the course of maturation are quantitated with this approach. Nüzhenide 11-methyl oleoside was the most abundant molecule in all three cultivars, with values of up to 42,976 mg/kg for Frantoio, 46,531 mg/kg for Moraiolo and 42,597 mg/kg for Leccino, followed by nüzhenide, with values of up to 32,112 mg/kg for Frantoio, 20,919 mg/kg for Moraiolo and 30,491 mg/kg for Leccino. These data indicated that the highest concentration of nüzhenide 11-methyl oleoside was in the Moraiolo samples, the same samples in which the concentration of nüzhenide was the lowest, indicating a different relative concentration of these two molecules in the olive seeds of the cultivars analyzed in this study. 

Concerning changes over the course of ripening, the concentration of nüzhenide 11-methyl oleoside increased in all three cultivars from the first to the second/third sampling date and then decreased, particularly at the last sampling date. Nüzhenide showed a trend similar to that of nüzhenide 11-methyl oleoside for Frantoio and Leccino, while in Moraiolo, it slightly decreased from the first to the fifth sampling date, with a final sharp decrease at the two last sampling points (Table 2A–C). Overall, a rather trend for Moraiolo with respect to Frantoio and Leccino was observed; this was also confirmed by the nüzhenide 11-methyl oleoside/nüzhenide ratio, which ranged from 1.20 to 1.49 for Frantoio, 1.39 to 1.93 for Leccino and 1.75 to 2.34 for Moraiolo. 

Based on our data, to obtain olive seed extracts with the highest concentration of nüzhenide 11-methyl oleoside, olives of the Moraiolo cultivar should be harvested. For extracts with the highest concentration in nüzhenide, olives of the Frantoio or Leccino cultivars should be chosen. In both the cases, the highest amounts were from fruits harvested in the first part of October.

As for total phenolic compounds, the highest content during the first five sampling dates was observed in Leccino cv, followed by Frantoio and Moraiolo. This higher content was mainly due to the concentrations of nüzhenide 11-methyl oleoside isomers, which were higher in Leccino than in Moraiolo or Frantoio. At the last two sampling dates, the decrease in the main phenols was sharper in Leccino than in Moraiolo or Frantoio; therefore, on these two dates, the cultivar with the highest phenolic concentration was Frantoio.

Concerning molecules other than nüzhenide and nüzhenide 11-methyl oleoside, isomers of these two molecules showed the highest concentrations, with values ranging from a minimum range of 434–699 mg/kg for nüzhenide isomer in Moraiolo (in agreement with the lowest concentration of nüzhenide in this cultivar) to a maximum of 5229–10,138 mg/kg for nüzhenide 11-methyl oleoside isomer 1 in Leccino. Concerning tyrosol derivatives, their concentration was higher in Frantoio and Leccino than in Moraiolo, while the opposite behavior was observed for salidroside oleoside, which is still a tyrosol derivative. It could be hypothesized that some cultivars accumulate tyrosol in certain forms while others do so in other forms. Ligstroside oleoside was only present in low amounts in the samples from the Leccino variety at the first four sampling dates; isomers of nüzhenide di-(11-methyl oleoside) were present in low concentrations, with slightly increasing trends, mainly for Frantoio and Leccino. Verbascoside showed the highest concentrations in the Frantoio cultivar, with values up to 2080 mg/kg, followed by Leccino (up to 1745 mg/kg), while in Moraiolo, the values ranged from 321 to 787 mg/kg.

**Figure 5:**
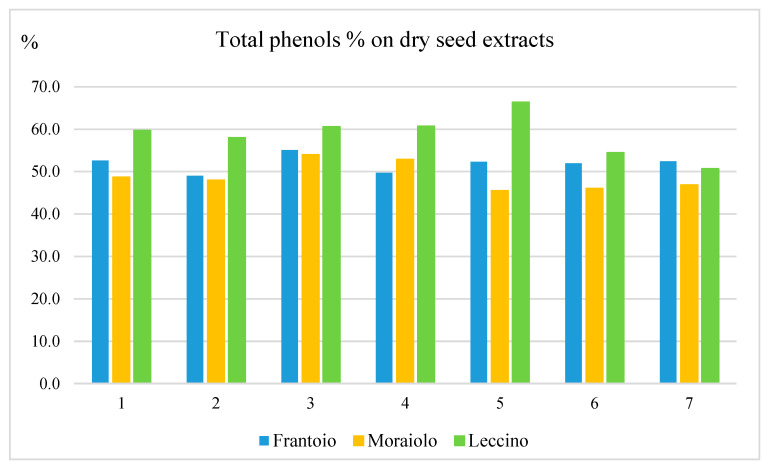
Percentage of total phenols in dry seed extracts for the three cultivars over the course of ripening. In the abscissa, the ripening period is indicated.

The data reported in Figure 5 highlight very high percentages of phenols in the dried seeds extracts, i.e., ranging from 45.5% for Moraiolo (M5) to 66.3% for Leccino (L5). These high phenolic percentages are driven by the abundance of two complex secoiridoids, i.e., as nüzhenide 11-methyl oleoside and nüzhenide, and by minor amounts of a number of other secoiridoids. 

### 2.4. Phenolic Profile of the Olive Pulp

As a final step of this work, we characterized the profile of the typical phenols present in olive pulp in the same samples used for seeds analysis. This step was aimed to complete the phenolic characterization of the collected samples, to compare the total content of phenols in the seeds with that of the typical phenols of the pulp and to compare the phenolic content of these samples with those analyzed over the course of ripening for the same cultivars in a previous study [27].

Table 3 reports the evolution over the course of ripening of the content of the molecules identified in olive pulps of the three cultivars, i.e., Frantoio (Table 3A), Moraiolo (Table 3B) and Leccino (Table 3C). Overall, 11 phenolic molecules were quantitated; oleuropein was by far the most abundant compound for the samples of the three cultivars. For Frantoio, the values decreased from 44,565 to 18,520 mg/kg over the course of ripening; for Moraiolo, the values increased from 23,056 mg/kg at the first sampling date to 40,597 mg/kg at the third, and then decreased to 33,262 mg/kg; for Leccino, this value ranged from 38,273 to 43,352 mg/kg in the first three sampling dates and then constantly decreased to 13,258 mg/kg. Overall, these values were slightly greater than those of the olive fruit samples harvested from the same field in a previous study [27]. Concerning the other molecules, the amounts were at least one order of magnitude lower than those of oleuropein over the entire ripening period, with only a few exceptions, as follows. Demethyloleuropein, which was initially absent, increased over time in fruit samples of the three cultivars, but with different trends: for the Frantoio cultivar, this compound was detected starting from the second sampling date; its abundance constantly increased thereafter, reaching values of 13,286 mg/kg, i.e., not much lower than those of oleuropein (18,520 mg/kg). For Moraiolo, this compound was detected only from the fifth sampling date, reaching values of up to 3610 mg/kg at the last sampling point, i.e., an order of magnitude lower than the value for oleuropein (33,262 mg/kg). In contrast, for Leccino, demethyloleuropein was detected starting from the third sampling date, reaching values of 21,053 mg/kg at the last sampling date, i.e., even higher than oleuropein (13,258 mg/kg) (Table 3A–C). This behavior is similar to what we observed in a previous study using samples from the same cultivars [27], confirming that demethyloleuropein is a degradation product of oleuropein, due to the action of endogenous esterase, and that it is cultivar dependent. Indeed, in both studies, the final values of demethyloleuropein were the lowest for Moraiolo and the greatest for Leccino. Another molecule that reached significant amounts was comselogoside. In the Frantoio samples, it decreased from 5112 mg/kg to 1223 mg/kg; in those of Leccino, it increased from 770 to 3721 mg/kg at the second sampling point and then constantly decreased to approx. 2000 mg/kg, while in the samples of Moraiolo, it increased from 1009 to 7677 mg/kg at the fourth sampling point and then decreased to 5376 (Table 3A–C). We do not want to speculate about the way this molecule is formed but, based on these data, we hypothesize that in the Moraiolo cultivar, it is likely synthesized via a diverse enzymatic activity.

Concerning the comparison among pulp and seed, the total concentration of phenols was comparable (Table 2 and Table 3), with values decreasing from 120,675 to 61,326 mg/kg in fruits of Frantoio and from 104,067 to 66,567 mg/kg in fruits of Leccino but increasing from 74,749 to 92,979 mg/kg at the fourth sampling date and then decreasing to 78,861 mg/kg at the end of sampling date in fruits of the Moraiolo cultivar. These findings disagree with previous works, which stated that phenolic compounds were present in the seeds in concentrations well below those in other tissues [6]. Our data also indicated that the content of the main phenolic compounds in the olive pulp (i.e., oleuropein) was comparable to those of nüzhenide and nüzhenide 11-methyl oleoside in the seeds of the same samples (Table 2). 

## 3. Materials and Methods

### 3.1. Chemicals

A Milli-Q-system (Millipore SA, Molsheim, France) was used to produce deionized water. Acetonitrile (HPLC-MS grade) was purchased from Panreac (Barcellona, Spain). Formic acid, hexane, methanol and ethanol were purchased from Merck (Darmstadt, Germany). The commercial standard of tyrosol was from Merck (Darmstadt, Germany), whereas those of oleuropein, nüzhenide, luteolin-7-*O*-glucoside, rutin, and verbascoside were from Extrasynthese Corporation (Genay, France).

### 3.2. Olive Fruit Samples Collection

Olive fruit samples of three typical Tuscan cultivars were harvested during ripening in the 2020 olive oil campaign (first sampling date: 9 September; last sampling date: 17 November).

For each of the three cultivars, 10 olive plants were selected at the Società Agricola Buonamici (Fiesole, Firenze) before the first sampling date. For each cultivar and for each of the sampling dates reported in Table 4, approx. 600 g of olive fruits were manually harvested from 10 selected plants. Olives were randomly harvested along the whole circumference of all the plants at a height close to 150–190 cm. Immediately after arriving in the laboratory, each olive sample was split into two aliquots, which were treated as described in the following paragraphs.

### 3.3. Pulp/Stone Ratio, Seed Yield and Lyophilization of the Whole Fruit

For each sample, a first aliquot of 100 olive fruits, randomly selected from the whole sample, was weighed. The olives were treated using a laboratory destoner (Toscana Enologica Mori, Tavarnelle Val di Pesa, Firenze, Italia), thus separating the stone from the pulp. The stones, which remained unbroken after pulp separation, were weighed, and the pulp/stone ratio was calculated as follows:

$$\frac{P}{S}=\frac{m_{100wf}+m_{100s}}{m_{100s}}$$

where *P*/*S* is the pulp/stone ratio, *m*_100*wf*_ is the mass of 100 whole fruits before destoning and *m*_100*s*_ is the mass of 100 stones.

In the following step, the 100 stones were lyophilized after freezing using liquid nitrogen. The lyophilized stones were then manually broken using a hammer, thus obtaining the olive seeds. The 100 seeds thus obtained were weighed and the percentage mass of the seeds with respect to the whole olive fruit was calculated. The seeds were then minced using a M20 Universal Mill (IKA-Werke Corporation, Staufen, Germany), and the obtained powder was used to prepare the phenolic extracts to be used for the HPLC-DAD-MS analysis as described in the following paragraphs.

A further aliquot of each olive fruit sample was lyophilized according to the method described in a previous study [27]. The lyophilized olives were crushed in a laboratory miller (Toscana Enologica Mori, Tavarnelle Val di Pesa, Firenze, Italia), and the obtained olive paste was used to characterize the phenolic composition of the olive samples, as described in the following paragraphs. 

### 3.4. Preparation of Phenolic Extracts from Olive Seeds

Phenolic compounds were extracted from olive seed powder using an extraction procedure suitable for both HPLC-DAD-MS analysis of phenolic compounds and for the preparation of dried extracts to be used for biological tests (which beyond the score of this manuscript) in the future steps of this study. 

Approx. 1 g of seed powder was added to 50 mL EtOH:H_2_O 90:10. The obtained mixture was first cold extracted with the aid of an Ultraturrax (2 min) and then with the aid of an ultrasound bath (20 min). Next, the mixture was centrifuged for 10 min at 20 °C and 5000 rpm. The supernatant was defatted twice with 30 mL of hexane and transferred into a 250-mL flask. The solvent was vacuum evaporated at 35 °C, and then the residue was recovered with three 2-mL aliquots of water with the aid of ultrasounds and transferred in a 10-mL volumetric flask, which was subsequently brought to volume. An aliquot of 1 mL of the solution was withdrawn, centrifuged for 4 min at room temperature and 14,000 rpm and immediately used for the HPLC-DAD-MS analysis. The remaining part (9 mL) was transferred into a previously weighed beaker, frozen at −20 °C and lyophilized for two days. The lyophilized extract was weighed to calculate the yield of seed phenolic extract. The dried extract was stored under vacuum for future biological assays (schematic in Figure 1).

### 3.5. Extraction of Phenolic Compounds from Lyophilized Olive Fruits

The olive pastes, obtained as described in Section 3.3, were used to characterize the phenolic composition of the whole olive fruit in order to make comparisons with the phenolic composition of olive fruits of the same cultivars from different crop seasons [27] but also to compare the abundances of phenolic compounds in the seed and the whole fruit.

Phenolic compounds were extracted from the olive fruit pastes using the following extraction procedure. Approx. 1 g of olive paste was cold extracted twice with 30 mL EtOH:H_2_O 80:20 by mixing for 4 min with an Ultraturrax. After each extraction cycle, the mixture was centrifuged for 10 min at 0 °C and 5000 rpm, and the supernatant was recovered. The obtained phenolic extract was defatted twice with 30 mL of hexane, then the hydroalcoholic solvent was evaporated under vacuum at 35 °C. The residue was recovered with 8 mL of MeOH:H_2_O 80:20 and the obtained suspension was centrifuged for 4 min at room temperature and 14,000 rpm in order to remove the insoluble residue. The supernatant was immediately used for the HPLC-DAD-MS analysis.

### 3.6. HPLC-DAD-MS Analysis of Phenolic Extracts

A chromatographic analysis of phenolic compounds extracted from both seeds and whole olives was performed using a previously described method [28] with slight modifications. The HPLC was a 1260 Infinity II LC System provided with two types of detectors: a Diode Array Detector (DAD) and a Mass Spectrometry Detector (MSD) equipped with an API-electrospray interface (InfinityLab LC/MSD) (both from Agilent, Santa Clara, CA, USA). The column was a Poroshell 120, EC-C18 (150 mm × 3.0 mm, 2.7 µm, from Agilent technology) which worked at a temperature of 26 °C and was safeguarded by a precolumn with the same stationary phase. The mobile phase was acetonitrile (A) and acidic H_2_O (formic acid, pH 3.2) (B). A multistep linear gradient was applied as follows: solvent A varied from 5% to 40% in the first 40 min, stayed at 40% for 5 min, then varied from 40% to 100% in 5 min; next, it stayed at 100% for three minutes before returning to 5% over 2 min, for a total analysis time of 55 min, followed by a post run reconditioning of 10 min. The flow rate was 0.4 mL min^−1^ and the injection volume was 2 µL. Chromatograms were recorded at 240, 280 and 350 nm. Regarding the MSD conditions, the ESI parameters were set as follows: nitrogen with a 10.5 L/min flow rate was used as a drying gas at a temperature of 350 °C; the pressure of the nebulizer was 1811 Torr; the capillary voltage was 3500 V. The acquisition was performed in an *m*/*z* range 150–2000 Th in full spectrum scan mode/negative ionization mode, applying the fragmentor at 200 V.

For the quantitative analysis, several calibration lines were built, using standards belonging to the chemical classes typical of the molecules identified in the analyzed phenolic extracts. In particular, they were: oleuropein (λ = 280 nm; linearity range 0–6.01 µg; R^2^ = 0.9985), nüzhenide (λ = 240 nm; 0–1.29 µg; R^2^ = 0.9999), luteolin-7-*O*-glucoside (λ = 280 nm; 0–2.79 µg; R^2^ = 0.9989), rutin (λ = 280 nm; 0–2.26 µg; R^2^ = 0.9997), tyrosol (λ = 280 nm; 0–1.22 µg; R^2^ = 1.0000) and verbascoside (λ = 280 nm; 0–1.98 µg; R^2^ = 0.9986). Tyrosol, hydroxytyrosol and their glycosylated derivatives were quantified using the calibration line of tyrosol and expressed as mg_tyr_/kg. Demethyloleuropein, ligstroside, oleuropein and their derivatives were quantified using the calibration line of oleuropein and expressed as mg_ole_/kg. Nüzhenide, nüzhenide 11-methyl oleoside and their isomers and derivatives were quantified with the calibration line of nüzhenide and expressed as mg_nuzh_/kg. Rutin was quantified using the calibration line of rutin and expressed as mg_rut_/kg. Luteolin-7-*O*-glucoside was quantified using the calibration line of luteolin-7-*O*-glucoside and expressed as mg_lut_/kg. Finally, verbascoside and comselogoside were quantified using the calibration line of verbascoside and expressed as mg_verba_/kg. All phenolic compounds were quantified at 280 nm with the exception of nüzhenide, nüzhenide 11-methyl oleoside and their isomers and derivatives, which do not absorb at 280 nm; consequently, these compounds were quantified at 240 nm.

### 3.7. Data Treatment

During the development phase of the method, one olive fruit sample and one olive seed sample (both constituted by a mixture of the available samples) were used to evaluate the precision, in terms of variability, of the quantitation of each phenolic compound in that matrix. To this end, the extraction and chromatographic analysis were repeated five times, and the obtained results were used to calculate the CV% of each phenol.

## 4. Conclusions

The work is a systematic study of olive seeds that sought to evaluate the biodiversity and the variability over time of the phenolic content in three cultivars. It provides, for the first time, the yields of the phenolic dry extracts of the seed and correlates the phenolic content in the pulp with that of the corresponding seed harvested at different ripening time. In all seed extracts, both nüzhenide and nüzhenide 11-methyl oleoside were consistently found to be the major phenolic compounds. Our results indicated that oleuropein, the main phenolic compound of the olive pulp, was present in amounts comparable to those of nüzhenide and nüzhenide 11-methyl oleoside in the seeds of the same samples. Knowledge of the correlation between the phenolic content in pulp and seed can help to evaluate the possibility of new uses of the whole fruit, and in particular, of the seed recovered from the pit, a by-product of the production of virgin olive oil, from de-stoned olives but also from pitted table olives. Finally, our study showed that the dried seed extracts are a rich source of total phenols, and in particular, of some complex secoiridoid compounds. 

## Figures and Tables

**Table 1:** For each of the collected olive fruit samples, the table reports the weights of 100 whole fruits and of the corresponding seeds, the percentage of the seed weight with respect to whole fruit, the pulp/stone ratio, the % yield of the phenolic extracts from seeds relative to the whole seed weight, and the fruit moisture content.

Sample	Sampling Date	Weight (g)		Seed % Weight	Pulp/Stone	Yield of Seed Phenolic Extract	Moisture Whole Fruit
		100 Whole Fruits	100 Seeds		Ratio	(% on Seed Weight)	(%)
F1	15 September 2020	101.5	2.195	2.16%	1.62	15.4%	44.7%
F2	6 October 2020	141.9	2.660	1.87%	1.97	19.5%	52.4%
F3	13 October 2020	149.2	3.345	2.24%	2.33	17.8%	52.9%
F4	20 October 2020	151.2	3.052	2.02%	2.24	17.2%	52.5%
F5	3 November 2020	144.7	3.248	2.24%	2.61	16.9%	53.1%
F6	10 November 2020	142.6	3.402	2.39%	2.64	16.5%	55.9%
F7	17 November 2020	166.6	4.323	2.59%	2.98	14.3%	57.6%
M1	15 September 2020	108.6	3.150	2.90%	2.38	15.9%	48.1%
M2	6 October 2020	133.4	3.395	2.54%	2.84	18.8%	53.3%
M3	13 October 2020	151.4	2.790	1.84%	3.48	16.2%	53.5%
M4	20 October 2020	160.8	3.035	1.89%	3.57	15.5%	53.0%
M5	3 November 2020	146.3	2.834	1.94%	3.53	17.6%	54.1%
M6	10 November 2020	159.2	3.362	2.11%	3.68	15.3%	53.1%
M7	17 November 2020	157.4	3.082	1.96%	3.95	13.4%	54.8%
L1	15 September 2020	106.0	2.515	2.37%	1.75	14.5%	48.0%
L2	6 October 2020	117.9	3.222	2.73%	1.88	17.1%	56.0%
L3	13 October 2020	120.2	3.405	2.83%	2.16	16.2%	58.5%
L4	20 October 2020	128.2	4.034	3.15%	1.94	15.0%	52.7%
L5	3 November 2020	139.9	4.154	2.97%	2.44	14.2%	58.7%
L6	10 November 2020	152.1	4.011	2.64%	2.61	13.3%	55.8%
L7	17 November 2020	136.2	4.418	3.24%	2.84	13.0%	56.8%

**Table 2:** Evolution of the content of phenolic compounds in seeds of olive fruits of the (A) Frantoio, (B) Moraiolo and (C) Leccino cultivars. Results are expressed in mg/kg of seed. The RSD was <5% (values determined as a mean of five replicates of a mixture of different samples).

A. FRANTOIO							
Phenolic Compound (mg/kg)	F1	F2	F3	F4	F5	F6	F7
tyrosol derivative 1	208	408	259	300	318	228	387
tyrosol derivative 2	392	776	498	575	305	238	200
tyrosol derivative 3	572	455	458	326	430	186	520
tyrosol derivative 4	313	1007	266	287	180	72	88
oleoside 11-methyl ester	605	1624	1193	1273	1024	678	760
oleoside 11-methyl ester isomer	1541	1101	970	649	606	628	358
nüzhnide derivative	923	1394	1449	1867	1545	1881	1123
verbascoside	823	2080	1187	1480	1552	1269	1290
nüzhnide	28,147	30,803	32,112	26,835	28,168	27,980	23,521
bis(oleoside 11-methyl ester) glycoside	1783	1222	1691	1580	1327	1625	1050
salidroside oleoside	1591	1786	2240	1867	1265	1501	985
nüzhnide isomer	2031	1792	1932	1093	1601	1557	1756
nüzhnide 11-methyl oleoside isomer 1	4489	6047	5889	3265	4378	3943	5264
nüzhnide 11-methyl oleoside isomer 2	2829	2360	3132	2012	2305	2396	2208
nüzhnide 11-methyl oleoside isomer 3	537	504	834	1148	1911	1311	2083
nüzhnide 11-methyl oleoside	33,742	41,639	42,976	39,942	40,156	39,190	31,286
nüzhnide di-(11-methyl oleoside) isomer 1	397	461	770	776	918	751	1423
nüzhnide di-(11-methyl oleoside) isomer 2	150	139	244	238	469	310	673
ligstroside oleoside	nd	nd	nd	nd	nd	nd	nd
nüzhenide 11-methyl oleoside/ nüzhenide ratio	1.1988	1.3518	1.3383	1.4884	1.4256	1.4006	1.3301
Total phenols	81,073	95,598	98,100	85,513	88,458	85,744	74,975
**B. MORAIOLO**							
**Phenolic Compound (mg/kg)**	**M1**	**M2**	**M3**	**M4**	**M5**	**M6**	**M7**
tyrosol derivative 1	269	498	440	491	302	160	174
tyrosol derivative 2	317	249	366	313	275	167	92
tyrosol derivative 3	273	252	284	185	244	157	174
tyrosol derivative 4	58	106	68	34	102	39	44
oleoside 11-methyl ester	1577	1485	2222	2251	1455	949	840
oleoside 11-methyl ester isomer	504	358	488	462	360	226	199
nüzhnide derivative	2159	2261	2602	2621	2722	2477	1882
verbascoside	321	657	716	787	593	404	428
nüzhnide	20,919	20,250	19,736	18,274	19,439	16,879	14,463
bis(oleoside 11-methyl ester) glycoside	2058	2842	2457	2255	2264	2128	1877
salidroside oleoside	2765	3401	3352	3120	2697	2148	2379
nüzhnide isomer	699	688	536	506	629	434	516
nüzhnide 11-methyl oleoside isomer 1	4987	5900	5202	4267	2554	2433	3351
nüzhnide 11-methyl oleoside isomer 2	1280	1487	1271	1015	1159	911	1269
nüzhnide 11-methyl oleoside isomer 3	1481	1861	1883	1558	1890	1656	1796
nüzhnide 11-methyl oleoside	36,590	46,531	44,407	42,683	42,155	38,070	31,916
nüzhnide di-(11-methyl oleoside) isomer 1	931	1165	1213	931	972	973	1164
nüzhnide di-(11-methyl oleoside) isomer 2	155	178	170	144	200	162	222
ligstroside oleoside	nd	nd	nd	nd	nd	nd	nd
nüzhenide 11-methyl oleoside/ nüzhenide ratio	1.749	2.298	2.250	2.336	2.169	2.255	2.207
Total phenols	77,343	90,169	87,413	81,897	80,012	70,373	62,786
**C. LECCINO**							
**Phenolic Compound (mg/kg)**	**L1**	**L2**	**L3**	**L4**	**L5**	**L6**	**L7**
tyrosol derivative 1	283	455	421	647	467	552	385
tyrosol derivative 2	345	655	468	624	208	433	269
tyrosol derivative 3	1481	891	458	595	433	471	341
tyrosol derivative 4	607	242	91	108	118	130	72
oleoside 11-methyl ester	1586	1510	1206	1190	1290	667	669
oleoside 11-methyl ester isomer	2523	1524	1103	971	713	506	320
nüzhnide derivative	341	946	1235	1430	1115	1907	1558
verbascoside	1267	1745	1353	1632	1384	1508	798
nüzhnide	24,359	30,491	29,355	25,836	29,693	18,678	16,294
bis(oleoside 11-methyl ester) glycoside	467	972	1382	933	1234	1535	1282
salidroside oleoside	908	1231	1161	1752	968	874	759
nüzhnide isomer	2101	2337	2215	1690	2231	1313	1086
nüzhnide 11-methyl oleoside isomer 1	8143	10,138	9340	5396	5819	6563	5229
nüzhnide 11-methyl oleoside isomer 2	3159	3512	3241	2991	2858	2530	2387
nüzhnide 11-methyl oleoside isomer 3	222	538	1120	1404	2322	1622	1539
nüzhnide 11-methyl oleoside	38,304	40,916	42,395	42,597	41,390	31,467	31,379
nüzhnide di-(11-methyl oleoside) isomer 1	153	643	1052	759	1251	1167	1048
nüzhnide di-(11-methyl oleoside) isomer 2	98	257	421	385	688	506	443
ligstroside oleoside	232	145	72	48	nd	nd	nd
nüzhenide 11-methyl oleoside/ nüzhenide ratio	1.572	1.342	1.444	1.649	1.394	1.685	1.926
Total phenols	86,579	99,148	98,089	90,988	94,182	72,429	65,858

**Table 3:** Evolution of the content of phenolic compounds in the pulp of whole lyophilized olive fruits of (A) Frantoio cultivar, (B) Moraiolo cultivar, (C) Leccino cultivar. Results are expressed in mg/kg of whole dried olives. The RSD was <5% (values determined as a mean of five replicates of a mixture of different samples).

(A)							
Phenolic Compound (mg/kg)	F1	F2	F3	F4	F5	F6	F7
hydroxytyrosol glucoside	141	119	-	-	-	-	-
hydroxytyrosol	461	211	185	210	242	274	191
tyrosol glucoside	41	56	111	82	265	198	163
tyrosol	35	116	128	136	96	188	166
demethyloleuropein	-	328	451	1060	9847	14,407	13,286
rutin	1003	683	505	642	695	599	484
luteolin-7-O-glucoside	634	366	297	380	539	379	382
verbascoside	5449	1477	1550	1022	1485	1309	890
oleuropein	44,565	28,243	28,097	21,296	33,358	20,152	18,520
comselogoside	5112	2329	2557	1028	2453	1592	1223
ligstroside	2925	1410	1777	1348	1203	832	695
total phenols	120,675	69,538	61,496	53,222	82,712	71,687	61,326
**(B)**							
**Phenolic Compound (mg/kg)**	**M1**	**M2**	**M3**	**M4**	**M5**	**M6**	**M7**
hydroxytyrosol glucoside	76	157	27	21	-	-	-
hydroxytyrosol	476	165	180	179	163	174	194
tyrosol glucoside	65	70	204	229	331	345	346
tyrosol	159	46	64	61	103	103	158
demethyloleuropein	-	-	-	-	966	3701	3610
rutin	695	936	800	903	1090	897	1037
luteolin-7-O-glucoside	294	480	405	484	562	462	527
verbascoside	2600	2166	3115	3206	1636	2094	1282
oleuropein	23,056	34,024	40,597	39,373	34,757	31,391	33,262
comselogoside	1009	5758	7300	7677	5519	6189	5376
ligstroside	1801	1474	2106	2061	1147	1398	1025
total phenols	74,749	83,492	91,901	92,979	80,026	81,513	78,861
**(C)**							
**Phenolic Compound (mg/kg)**	**L1**	**L2**	**L3**	**L4**	**L5**	**L6**	**L7**
hydroxytyrosol glucoside	188	219	-	-	-	-	-
hydroxytyrosol	1179	470	84	49	140	75	95
tyrosol glucoside	222	194	181	150	164	219	196
tyrosol	146	99	240	224	218	352	304
demethyloleuropein	-	-	1787	2574	8920	17,445	21,053
rutin	868	988	816	831	716	454	575
luteolin-7-O-glucoside	218	328	340	309	362	203	319
verbascoside	571	483	837	305	447	293	322
oleuropein	38,273	42,176	43,352	30,006	37,337	20,734	13,258
comselogoside	770	3721	2658	1588	1740	1810	2042
ligstroside	1861	1512	1555	1261	1428	622	302
total phenols	104,067	87,215	84,480	69,952	82,216	77,287	66,567

**Table 4:** The olive fruit samples of three Tuscan cultivars collected over the course of ripening in the 2020 crop season.

*Sampling Date*	Frantoio cv	Moraiolo cv	Leccino cv
15 September 2020	F1	M1	L1
6 October 2020	F2	M2	L2
13 October 2020	F3	M3	L3
20 October 2020	F4	M4	L4
3 November 2020	F5	M5	L5
10 November 2020	F6	M6	L6
17 November 2020	F7	M7	L7

## Data Availability

Not applicable.

## Sample Availability

Samples of the compounds are not available from the authors.
